# Fabrication of Smart Chemical Sensors Based on Transition-Doped-Semiconductor Nanostructure Materials with µ-Chips

**DOI:** 10.1371/journal.pone.0085036

**Published:** 2014-01-13

**Authors:** Mohammed M. Rahman, Sher Bahadar Khan, Abdullah M. Asiri

**Affiliations:** 1 Center of Excellence for Advanced Materials Research, King Abdulaziz University, Jeddah, Saudi Arabia; 2 Chemistry Department, Faculty of Science, King Abdulaziz University, Jeddah, Saudi Arabia; RMIT University, Australia

## Abstract

Transition metal doped semiconductor nanostructure materials (Sb_2_O_3_ doped ZnO microflowers, MFs) are deposited onto tiny µ-chip (surface area, ∼0.02217 cm^2^) to fabricate a smart chemical sensor for toxic ethanol in phosphate buffer solution (0.1 M PBS). The fabricated chemi-sensor is also exhibited higher sensitivity, large-dynamic concentration ranges, long-term stability, and improved electrochemical performances towards ethanol. The calibration plot is linear (r^2^ = 0.9989) over the large ethanol concentration ranges (0.17 mM to 0.85 M). The sensitivity and detection limit is ∼5.845 µAcm^−2^mM^−1^ and ∼0.11±0.02 mM (signal-to-noise ratio, at a SNR of 3) respectively. Here, doped MFs are prepared by a wet-chemical process using reducing agents in alkaline medium, which characterized by UV/vis., FT-IR, Raman, X-ray photoelectron spectroscopy (XPS), powder X-ray diffraction (XRD), and field-emission scanning electron microscopy (FE-SEM) etc. The fabricated ethanol chemical sensor using Sb_2_O_3_-ZnO MFs is simple, reliable, low-sample volume (<70.0 µL), easy of integration, high sensitivity, and excellent stability for the fabrication of efficient I–V sensors on μ-chips.

## Introduction

The development of chemi-sensors has been the subject of considerable interest in recent years based on μ-chips using specific matrixes. Such modification on μ-chip offers great attention to chemical detection that included high-sensitivity, inherent-miniaturization, low-cost, high selectivity, low-sample volume, independence of sample turbidity or optical-path length, minimal-power demands, and high bio-compatibility with advanced micro-fabrication technologies. The development of electrochemical chemi-sensors for sensitive detection of toxic chemicals is generally required innovative approaches that coupled with various modification/amplification procedures onto electro-active substrates. Semiconductor codoped materials have attracted much interest because of their unique properties and potential applications in all areas of advance science and technological fields [1]. The simplest synthetic route for doped-materials is possibly self-aggregation, in which ordered doped aggregates are prepared in economical approaches [2]. Although, it is still a big-challenge to develop simple and economical route for microstructures semiconductor codoped metal oxide with designed chemical components and controlled morphologies, which is strongly influenced the optical and electrical properties of doped nanomaterials [3]. The significance of safety for human-beings and environments has been considered with great attention in doped semiconductor chemi-sensors for toxic chemical detection (toxic level in human blood, >0.10% or 2.2 mM) by reliable methods using μ-chips [4], [5]. Semiconductor micro-structure materials are very sensitive due to their particle-size and high-active surface-area as compared to the transition materials in micro-ranges. Nanoscale materials composed micro-structures have also displayed a huge-deal of consideration due to their promising properties such as large active-surface area, high-stability, quantum confinement, high-porosity, and permeability (meso-porous nature), which is directly dependent on the shape and size of the microcrystals [6], [7]. During the last two decades, semiconductor nanomaterials have been received significant attention due to their electronic, magnetic, electrical, optoelectronic, mechanical properties, and their prospective applications in nanotechnology fields. Doped materials might be a promising candidate because of their high-specific surface-area, low-resistance, fascinating electrochemical, and optical properties [8], [9]. Solution–liquid–solid mechanism [10], vapor–solid mechanism [11], and oxide-assisted growth mechanism [12] have also been adopted to prepare various antimony oxide nanostructure materials. Recently, antimony oxide nanostructure have been also prepared using micro-emulsion [13] and templating CNTs [14] by several groups, however, nanosheets composed microstructure of Sb_2_O_3_-doped ZnO MFs have never reported. In this report, it is displayed an alternative approach to the synthesis of Sb_2_O_3_-ZnO MFs with diameters of several nanometers sheet composed several micrometers flowers by a wet-chemical process. Here, semiconductor zinc oxide (ZnO, band-gap ∼3.4 eV, II–VI compound, binding energy ∼60 meV, excellent acoustic wave, and n-type semiconductor) has been recognized as a promising host material at room conditions, which displayed a wurtzite type structure with number of periodic planes with hexagonally-coordinated O and Zn atoms piled along the c-axis [15]. For outstanding and extraordinary properties of ZnO, it is used for flexible applications in piezo-electric chips, opto-electronics, photo-catalytic, solar cells, transparent thin- film transistors, bio- and chemi-sensors, spintronics, light-emitters, electronics, catalysis, and so forth [16]–[18]. For exotic and flexible properties including bio-compatibility, non-toxicity, chemical and photo-chemical stability, high-specific surface area, optical and electro-chemical behaviors, and high-electron communication characteristics, the transition-doped semiconductor nanomaterials presents itself as one of the most promising materials for the development and fabrication of efficient chemi-sensors [19], [20]. Recently, the extensive progresses have been explored on ZnO-based nanomaterials synthesis by a wet-chemical and conventional techniques [21]. Zinc oxide nanostructure displayed attractive applications, such as transistors, UV photo-detectors, gas-sensors, field-emission electron sources, nano-wires and nano-lasers, nano-scale power generators, and many other functional devices [22]–[27].

Advances in nanotechnology for innovative chemi-sensors, nanomaterials embedded μ-chips have been regulating a key-task in the fabrication and improvement of very precise, perceptive, accurate, sensitive, and consistent sensors. The exploration for even tiny chips accomplished in nano-level imaging and controlling of doped nanomaterial for biological, chemical, pathological samples, chemi-sensor has recently expanded the spotlight of awareness of scientist mainly for control monitoring due to the amplifying essential for environmental safety and health monitoring [28], [29]. Transition-doped semiconductor metal oxides are the model materials for sensing due to high-active surface areas and extensively employed as sensor for the detection, recognition, and quantification of various toxic pollutants and hazardous chemicals [30]–[34]. In presence of ethanol, it causes damage of brain and specific diseases of stomach, liver, and erythrocyte. Therefore it is important and big challenge to sense ethanol efficiently and shield the human health from dangerous diseases and safe the environment using electro-analytical methods [35]. Recently, a large amount of undoped metal oxides is considered as chemical sensors for the detection of different hazardous pollutants and toxic chemicals [36], [37]. Therefore Sb_2_O_3_-ZnO MFs have been offered as a mediator to detect and quantify the ethanol chemicals in liquid phase. The main attention of the present investigation is to fabricate and develop a highly sensitive chemi-sensor (especially ethanol) for detecting and quantifying hazardous pollutants using Sb_2_O_3_-ZnO MFs on μ-chips. Hence a well-crystalline Sb_2_O_3_-ZnO MFs were prepared by a wet-chemical process and totally characterized by using UV/visible, FT-IR spectroscopy, XRD, FESEM, and XPS analysis etc.

In this contribution, it is prepared by a wet-chemical process to arrange as-prepared Sb_2_O_3_ doped ZnO MFs with practically controlled flower-shape structures, which exposed a control-morphological development in microstructure materials and potential chemi-sensor applications using tiny μ-chips. With most of the significant properties of the doped material, there have been more and more attention focused to explore the doped counterparts. For semiconductor materials, doping is an exceptional function to improve significantly the optical and electrical properties, which enhances the development of electronic and opto-electronic devices. Codoped nanosheets composed Sb_2_O_3_-ZnO MFs are used to fabricate by a simple hydrothermal method on a tiny μ-chip surfaces, which allows very sensitive transduction of liquid/surface interactions and measured the chemical sensing performance considering ethanol at ambient conditions. To best of our knowledge, this is the first report for detection of toxic ethanol with as-grown Sb_2_O_3_-ZnO MFs onto tiny μ-chips using reliable I-V method in short response time.

## Experimental Sections

### Materials and Methods

Zinc chloride, butyl carbitol acetate, antimony chloride, ethyl acetate, ammonia solution (25%), and all other chemicals were in analytical grade and purchased from Sigma-Aldrich Company. They were used without further purification. The λ_max_ (289.0 nm) of as-grown Sb_2_O_3_ doped ZnO MFs was executed using UV/visible spectroscopy Lamda-950, Perkin Elmer, Germany. FT-IR spectra of MFs were measured on a spectrum-100 FT-IR spectrophotometer in the mid-IR range purchased from Bruker (ALPHA, USA). The XPS measurements of MFs were executed on a Thermo Scientific K-Alpha KA1066 spectrometer (Germany). Monochromatic AlK_α_ x-ray radiation sources were used as excitation sources, where beam-spot size was kept in 300.0 µm. The spectrum was recorded in the fixed analyzer transmission mode (pass energy, ∼200.0 eV), where the sample scanning of the spectra was performed less 10^−8^ Torr. Morphology, size, elemental, and structure evaluation of as-grown MFs were recorded on FE-SEM instrument from JEOL (JSM-7600F, Japan). The powder XRD patterns of MFs were recorded by X-ray diffractometer from PANalytical diffractometer equipped with Cu-K_α1_ radiation (*λ* = 1.5406 nm). The generator voltage (∼45.0 kV) and generator current (∼40.0 mA) were applied for the XRD measurement. Raman spectrometer was used to measure the Raman shift of as-grown MFs using radiation source (Ar^+^ laser line, *λ*; 513.4 nm), which was purchased from Perkin Elmer (Raman station 400, Perkin Elmer, Germany). I–V technique (two electrodes composed on μ-chip) is measured by using Keithley-Electrometer from USA.

### Preparation and growth mechanism of Sb_2_O_3_ doped ZnO MFs

Large-scale synthesis of Sb_2_O_3_-ZnO MFs was prepared by a wet-chemical process at low-temperature using zinc chloride (ZnCl_2_), antimony chloride (SbCl_3_), and ammonium hydroxide (NH_4_OH). In a usual reaction process, 0.1 M ZnCl_2_ dissolved in 50.0 ml deionized (DI) water mixed with 50.0 ml DI solution of 0.1 M SbCl_3_ and 50.0 ml of 0.1 M urea under continuous stirring. The pH of the solution was adjusted to 9.7 by addition of NH_4_OH and resulting mixture was shacked and stirred continuously for 30.0 minutes at room conditions. After stirring, the solution mixture was then put into conical flux and heat-up at 160°C for 12.0 hours. The temperature of solution was controlled manually throughout the reaction process at 90.0°C. After heating the reactant mixtures, the flux was kept for cooling at room conditions until reached to room temperature. The final codoped products were obtained, which was washed thoroughly with DI water, ethanol, and acetone for several times subsequently and dried at room-temperature for structural, elemental, morphological, and optical characterizations. The growth mechanism of the Sb_2_O_3_-ZnO nanostructure materials can be explained on the basis of chemical reactions and nucleation as well as growth of doped nanocrystals. The probable reaction mechanisms are presented here for attaining the codoped nanomaterial oxides in below. 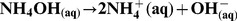
(i)

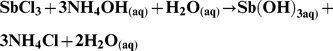
(ii)

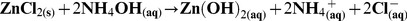
(iii)

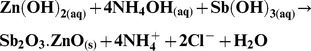
(iv)


The reaction is forwarded slowly according to the proposed equation (i) to equation (iii). During preparation, the pH value of the reaction medium plays an important responsibility in the doped nano-material oxide formation. At a particular pH, when SbCl_3_ is hydrolyzed with ammonia solution, antimony hydroxide is formed instantly according to the equation (ii). During the whole synthesis route, NH_4_OH operates a pH buffer to control the pH value of the solution and slow contribute of hydroxyl ions (OH^-^). When the concentrations of the Sb^3+^ and OH^−^ ions are achieved above in critical value, the precipitation of Sb_2_O_3_ nuclei begin to start. As there is high concentration of Zn^2+^ ions in the solution [according to the reactions (iii)], the nucleation of Sb_2_O_3_ crystals become slower due to the lower activation energy barrier of heterogeneous nucleation. Hence, as the concentration of Zn^2+^ existences, a number of larger Sb_2_O_3_.ZnO crystals with aggregated nanosheets composed flower-like morphology form after the reactions [equation (v)]. The shape of calcined Sb_2_O_3_.ZnO MFs is approximately reliable with the growth pattern of antimony doped zinc oxides crystals [38]–[40]. Then the solution was washed thoroughly with acetone, ethanol, and water consecutively and kept for drying at room condition. Finally, the as-grown doped Sb_2_O_3_.ZnO MFs nanomaterials were calcined at 400.0°C for 6 hours in the furnace (Barnstead Thermolyne, 6000 Furnace, USA). The calcined MFs were characterized in detail in terms of their morphological, structural, optical properties, and applied for ethanol chemical sensing based on μ-chips for the first time.

### Fabrication and detection technique of ethanol using Sb_2_O_3_-ZnO MFs on μ-chips

The sensing area of μ-chip is fabricated with as-grown Sb_2_O_3_ doped ZnO MFs using butyl carbitol acetate (BCA) and ethyl acetate (EA) as a conducting coating agent by drop-coating method. Then it is put into oven at 60.0°C for two hours until the film is completely dry. 0.1 M phosphate buffer solution (PBS) at pH 7.0 is made by mixing 0.2 M Na_2_HPO_4_ and 0.2 M NaH_2_PO_4_ solution in 100.0 mL de-ionize water. A cell is consisted of Sb_2_O_3_-ZnO MFs fabricated μ-chip as a working and Pt connector is used a counter electrodes. As received ethanol is diluted to make various concentrations (0.17 mM to 8.5 M) in PBS solution and used as a target analyte. 70.0 µL of 0.17 mM PBS solution is kept constant during measurements onto the μ-chip. The ratio of current versus concentration (slope of calibration curve) is used to calculate of target ethanol chemical sensitivity. Limit of detection (LOD) is calculated from the ratio of 3N/S versus sensitivity (ratio of noise×3 vs. sensitivity) in the linear dynamic ranges of calibration plot. Electrometer is used as a voltage sources for I–V measurement in simple two electrodes μ-chip system. The as-grown Sb_2_O_3_-ZnO MFs are fabricated and employed for the detection of target analyte.

## Results and Discussion

### Optical properties of Sb_2_O_3_ doped ZnO MFs

**Figure 1:**
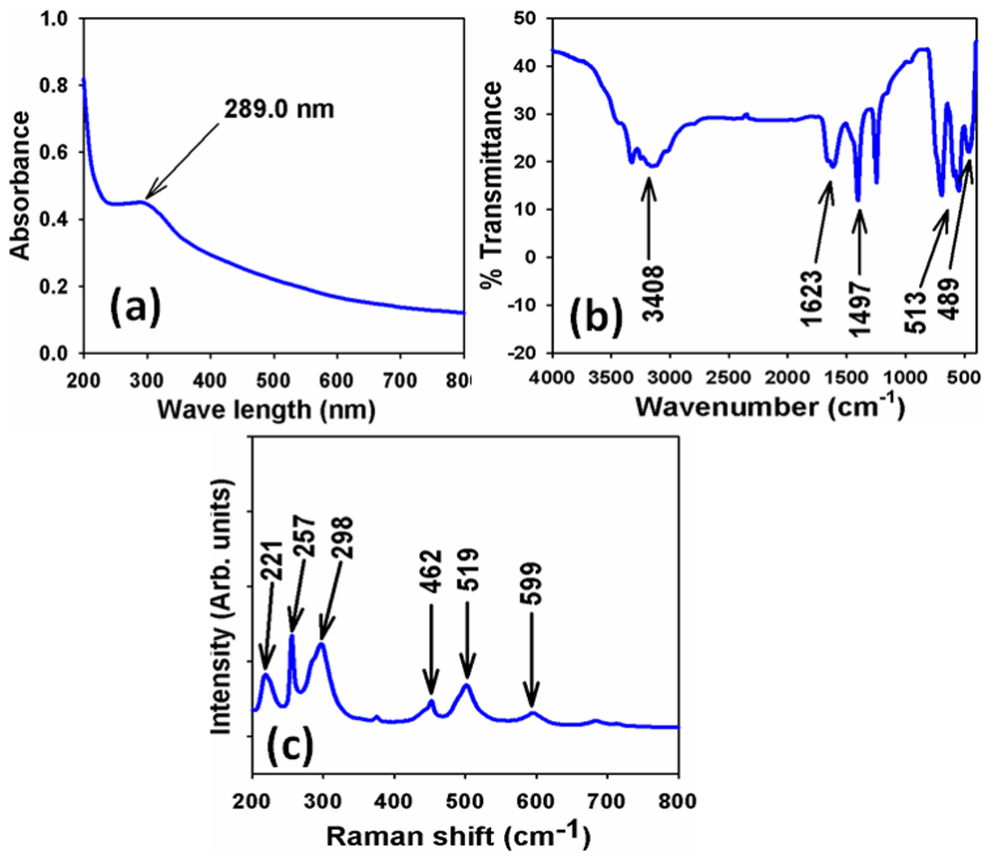
(a) UV/visible, (b) FT-IR, and (c) Raman spectroscopy of as-grown Sb_2_O_3_ doped ZnO MFs at room conditions.



The optical property of the as-grown Sb_2_O_3_ doped ZnO MFs is one of the significant characteristics for the assessment of its photo-catalytic activity. UV/visible absorption is a technique in which the outer electrons of atoms or molecules absorb radiant energy and undergo transitions to high energy levels. In this phenomenon, the spectrum obtained due to optical absorption can be analyzed to acquire the energy band-gap of the doped metal oxides. For UV/visible spectroscopy, the absorption spectrum of Sb_2_O_3_-ZnO MFs solution is measured as a function of wavelength, which is presented in Figure 1A. It presents a broad absorption band around 289.0 nm in the visible-range between 200.0 to 800.0 nm wavelengths indicating the formation of Sb_2_O_3_-ZnO MFs. Band-gap energy (E_bg_) is calculated on the basis of the maximum absorption band of Sb_2_O_3_-ZnO MFs and found to be ∼4.2907 eV, according to following equation (v).

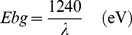
(v)Where *E*_bg_ is the band-gap energy and λ_max_ is the wavelength (289.0 nm) of the Sb_2_O_3_-ZnO MFs. No extra peak associated with impurities and structural defects are observed in the spectrums, which proved that the synthesized microstructure controlled crystallinity of as-grown Sb_2_O_3_-ZnO MFs [41], [42].

The as-grown Sb_2_O_3_ doped ZnO MFs is also investigated in terms of the atomic and molecular vibrations. To predict the functional-recognition, FT-IR spectra fundamentally in the region of 400∼4000 cm^−1^ is investigated at room conditions. Figure 1B displays the FT-IR spectrum of MFs, which represents band at 489, 513, 1497, 1623, and 3408 cm^−1^. These observed broad vibration bands (at 489 & 513 cm^−1^) could be assigned as metal-oxygen (Sb-O & Zn-O mode) stretching vibrations, which demonstrated the configuration of Sb_2_O_3_-ZnO MF materials. The supplementary vibrational bands may be assigned to O-H bending vibration, C-O absorption, and O-H stretching. The absorption bands at 1497, 1623, and 3408 cm^−1^ generally shows from CO_2_ and water, which usually semiconductor doped nanostructure materials absorbed from the environment due to their high surface-to-volume ratio of mesoporous nature [43], [44]. Finally, the experimental vibration bands at low frequencies regions recommended the formation of Sb_2_O_3_-ZnO MFs by a facile wet-chemical method. Raman spectroscopy is a spectroscopic technique utilized to display vibrational, rotational, and other low-frequency phases in a Raman active compound. It depends on inelastic scattering of monochromatic light (Raman scattering), usually from a laser in the visible, near infra-red, or near ultra-violet range. The laser light relates with molecular vibrations, phonons or other excitation in the modes, showing in the energy of the laser photons being shifted up or down. The shift in energy represents the information regarding the phonon modes in the system, where infrared spectroscopy yields similar, but complementary information. Raman spectroscopy is generally established and utilized in material chemistry, since the information is specific to the chemical bonds and symmetry of metal-oxygen stretching or vibrational modes. Usually, there are three vibration modes in Sb_2_O_3_-ZnO MFs nanomaterial crystal: A_1_, E_1_ and E_2_, of which A_1_ and E_1_ split into longitudinal (A_1L_, E_1L_) and transverse (A_1T_, E_1T_) ones and E_2_ contains low and high frequency phonons (E_2L_ and E_2H_) [45], [46]. As-grown Sb_2_O_3_-ZnO MFs is significantly altered the Raman spectra as well as the crystal of ZnO nanostructure [47], [48]. Here, Figure 1c confirms the Raman spectrum, where key aspects of the wave number are employed at about 221, 298, and 257 cm^−1^ for metal-oxygen (Sb-O and Zn-O) stretching vibrations. The large bands can be assigned to a cubic phase of Sb_2_O_3_-ZnO MFs. At 462, 519, and 599 cm^−1^ higher wave-number shifts are revealed owing to the different dimensional effects of the MFs.

### Morphological, Structural, and Elemental properties of Sb_2_O_3_ doped ZnO MFs

**Figure 2:**
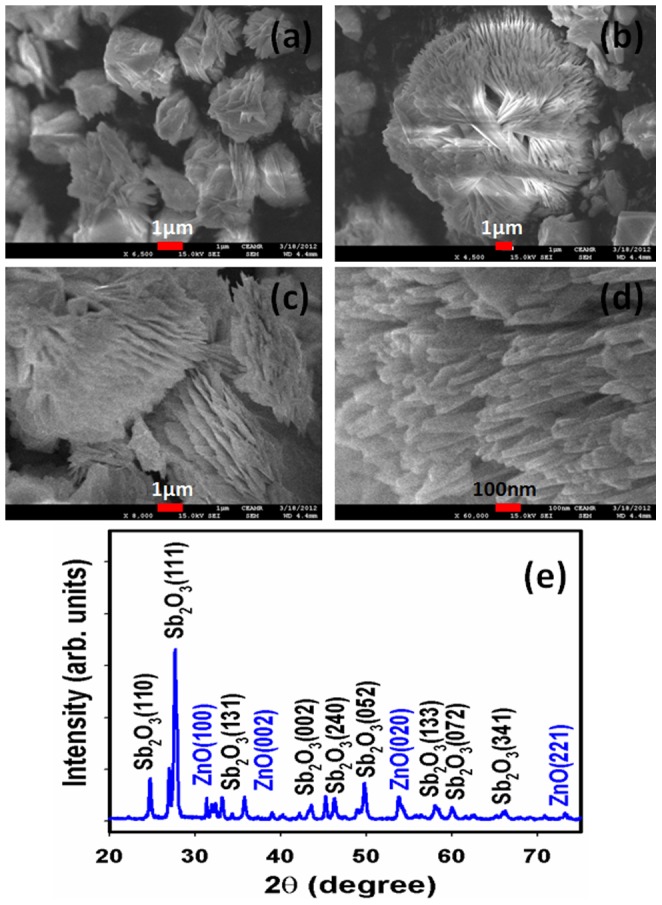
(a–d) FE-SEM images and (e) Powder x-ray diffraction pattern, of as-grown Sb_2_O_3_-ZnO MFs at room conditions.

FE-SEM images of as-grown Sb_2_O_3_ doped ZnO MFs structures are presented in Figure 2(a–d). It exhibits the images of the MFs with micro-dimensional sizes of as-grown Sb_2_O_3_-ZnO MFs. The dimension of MF is calculated in the range of 2.4 µm, which composed of nanosheets (∼65.0±10.0 nm). It is clearly exposed from the FE-SEM images that the facile synthesized Sb_2_O_3_-ZnO MFs is microstructures in flower-shape, which is grown in very high-density and possessing almost uniform nanosheet composed MFs. When the size of doped material decreases into micrometer-sized scale, the surface area is increased significantly, this improved the energy of the system and made re-distribution of Zn and Sb ions possible. The micrometer-sized flower could have tightly packed into the lattice, which is an agreement with the publish reports [49], [50]. Crystallinity and crystal phase of Sb_2_O_3_-ZnO MFs were investigated using by powder X-ray diffractometer. Powder X-ray diffraction patterns of doped MFs are represented in Figure 2e. The Sb_2_O_3_.ZnO MFs were investigated and exhibited as face-centered cubic shapes. Figure 2e reveals characteristic crystallinity of the codoped Sb_2_O_3_.ZnO MFs and their crystalline arrangement, which is investigated using powder X-ray crystallography. All the reflection peaks in this prototype were related with ZnO phase having face-centered cubic zincite geometry [JCPDS # 071-6424]. The phases demonstrated the key features with indices for crystalline ZnO at 2θ values of 32.3(001), 39.8(002), 54.7(020), and 73.2(221) degrees. The face-centered cubic lattice parameters are a = 3.2494, b = 5.2038, and radiation (CuK_α_1, λ = 1.5406). The ZnO phases have a high degree of crystallinity. All of the peaks match well with Bragg reflections of the standard zincite structure (point or space-group P63mc) [51]–[53]. The reflected peaks were also found to correspond with Sb_2_O_3_ phase having face-centered cubic orthorhombic geometry [JCPDS # 074-1725]. The phases demonstrated the key features with indices for crystalline Sb_2_O_3_ at 2θ values of 25.7(110), 28.1(111), 32.3(131), 43.6(002), 46.2(242), 59.2(052), 58.3(133), 60.3(072), and 67.4(341) degrees. The Sb_2_O_3_ phases have a high degree of crystallinity. All of the peaks match well with Bragg reflections of the standard orthorhombic structure. These confirmed that there is major number and amount of crystalline codoped Sb_2_O_3_-ZnO present in MFs [54].

**Figure 3:**
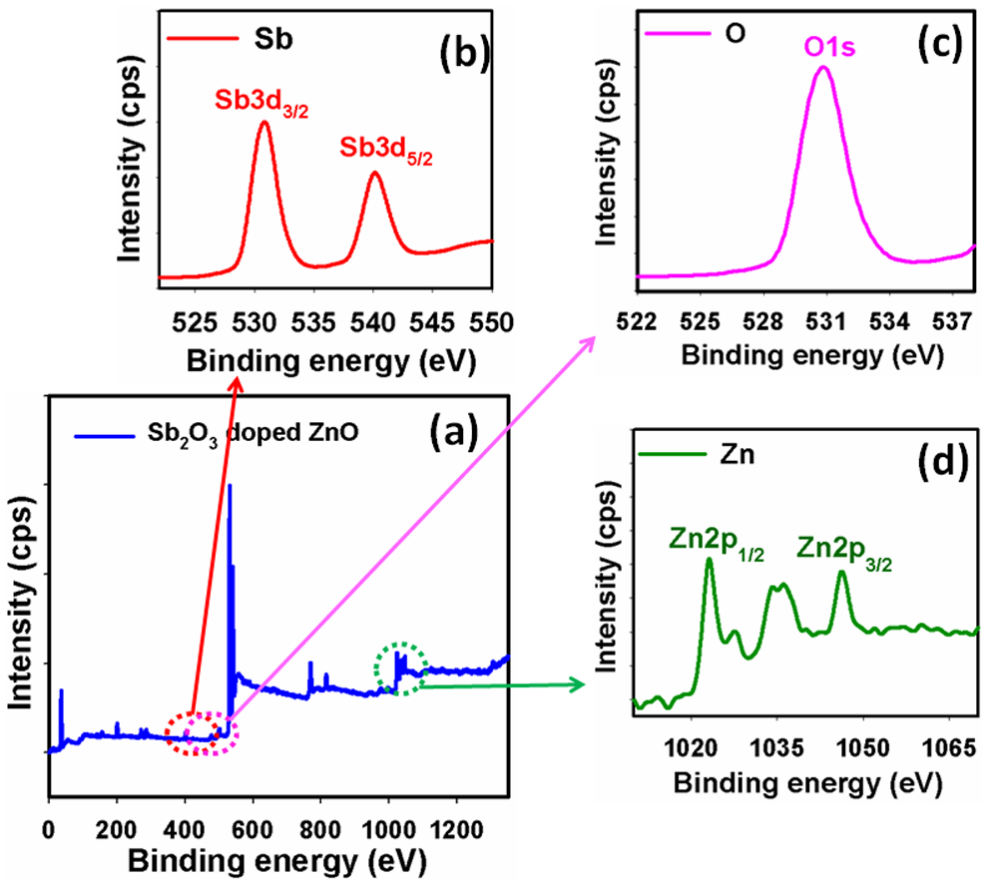
XPS of (a) doped Sb_2_O_3_-ZnO MFs, (b) Sb3d level, (c) O1s level, and (d) Zn2p level acquired with MgKα1 radiation.

X-ray photoelectron spectroscopy (XPS) is a quantitative spectroscopic method that determines the chemical-states of the elements that present within doped materials. XPS spectra are acquired by irradiating on a nanomaterial with a beam of X-rays, while simultaneously determining the kinetic energy and number of electrons that get-away from the top one to ten nm of the material being analyzed. Here, XPS measurements were measured for Sb_2_O_3_-ZnO MFs semiconductor nanomaterials to investigate the chemical states of ZnO and Sb_2_O_3_. The XPS spectra of Sb3d, Zn2p, and O1s are presented in Fig. 3a. XPS was also used to resolve the chemical state of the doped Sb_2_O_3_ nanomaterial and their depth. Figure 3b presents the XPS spectra (spin orbit doublet peaks) of the Sb3d_(3/2)_ and Sb3d_(1/2)_ regions recorded with semiconductor doped materials. The binding energy of the Sb3d_(3*/*2)_ and Sb3d_(1/2)_ peak at 529.1 eV and 539.6 eV respectively denotes the presence of Sb_2_O_3_ since their bindings energies are similar [55]. The O1s spectrum shows a main peak at 531.2 eV in Fig. 3c. The peak at 531.2 eV is assigned to lattice oxygen may be indicated to oxygen (ie, O_2_^−^) presence in the doped Sb_2_O_3_-ZnO MF nanomaterials [56]. In Figure 3d, the spin orbit peaks of the Zn2p_(1/2)_ and Zn2p_(3/2)_ binding energy for all the samples appeared at around 1025 eV and 1048 eV respectively, which is in good agreement with the reference data for ZnO [57].

### Preparation of μ-Chips using photolithography method

**Figure 4:**
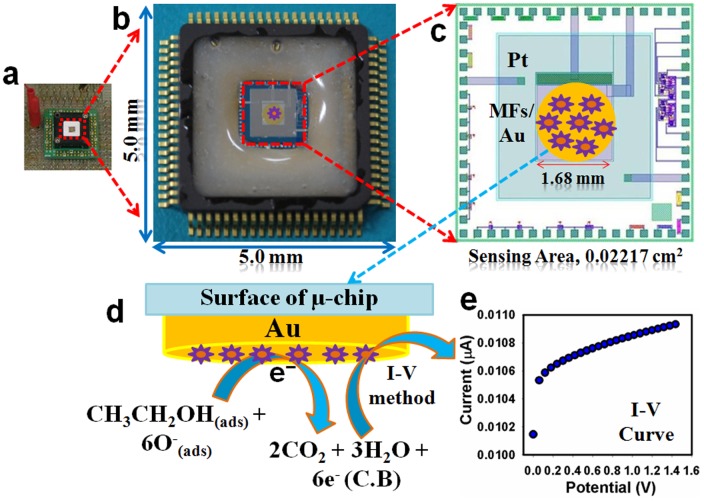
Schematic diagram of (a) real camera-view from top, (b) magnified view of μ-chip, (c) fabrication with Sb_2_O_3_-ZnO MFs with conducting binders (EC & BCA) onto μ-chip sensing-area, (d) reaction mechanism of ethanol in presence of doped Sb_2_O_3_-ZnO MFs, and (e) outcomes of I-V experimental results.

Electrochemical μ-chips were fabricated by conventional photolithographic technique, where electrodes and passivation layers are developed on silicon wafer followed by dicing and packaging [58]. Nitrogen-doped silicon wafers are prepared and overflowed by extra-pure water. In this step, all contaminations on the surface and native SiO_2_ layer are removed perfectly. At first, the wet oxidation is employed and then dry oxidation is executed, where, wafers are annealed in the nitrogen environment. Aluminum is sputtered with aluminum-1% Si target. Then the photolithograph processes are applied. Resist coating, baking, exposure, and development are employed by Kanto chemicals, and then it is rinsed thoroughly by ionic water. Aluminum is etched by etching solution and resistance layer is removed perfectly by plasma etching instrument. Then silicon wafers are cleaned by acetone, methanol, and finally by plasma simultaneously. Silicon nitride (SiN) layer is deposited by chemical vapor deposition and then pad electrode surfaces are etched by reactive ion etching. Finally residual resist layer is removed by plasma etching. After photolithographic process, platinum is sputtered by SP150-HTS. Then it is patterned by lift-off method, in which wafers are immersed into the remover, and then washed with isopropyl alcohol. Photolithographic process is again investigated, where titanium is sputtered as a binding layer, and then gold is evaporated by deposition method. Finally, gold layer is patterned by lift-off method. Palylene passivation layer is formed for the protection of the μ-chip from water. Photolithographic process is performed again for pad protection. Then palylene-dimer is evaporated by deposition apparatus. Photolithography process is done again for patterning. Palylene layer is patterned by etching. Finally, un-necessary resists are removed by acetone and then wafer is cleaned by isopropyl alcohol (IPA). Resist is coated on a whole surface of the silicon wafer for protection during dicing process is executed. Silicon wafer is diced into pieces by dicing apparatus and stored into the desiccators, when not in use. Resist on μ-chip surface is removed by acetone and cleaned with isopropyl alcohol (IPA). The opposite side of the chip is roughed by a sandpaper sheet for better adhesion and electrical stability. The μ-chip is bonded with die and packaged by silver paste. It is dried in a drying oven. Pads on chip are connected to the package through gold wire with bonding machine. Finally, silicon-based adhesive is put on the periphery of the chip to protect pads and gold wire from sample solution. Adhesive is dried for 24 hours at room temperature. The semiconductor smart μ-chips were fabricated on silicon wafer. Aluminum was sputtered to fabricate as wiring and bonding pads. Pt-Ti-TiN was sputtered on thermal oxide of silicon and patterned by photolithography to fabricate counter electrode (CE). Ti-TiN layers were used for strong adhesion. Au-Ti were sputtered and lithographed, which made circular working electrode (WE) with a diameter of 1.68 mm in the center of the μ-chip. After electrodes fabrication, palylene layer was fabricated by evaporation method as a passivation layer. The wafer was diced to 5.0 mm square μ-chips. This μ-chip was bonded to a package by silver paste. Aluminum pads were connected to the package by gold wire. Finally, adhesive (Araldite, Hantsman, Japan) was put on the periphery of the chip, which prevents target solution from contacting pads (Figure 4a). The magnified construction view of internal μ-chip center (sensing area) is presented in the Figure 4(b–c).

### Fabrication and chemical sensor application of Sb_2_O_3_-ZnO/μ-Chips assembly

The potential application of Sb_2_O_3_-ZnO MFs assembled onto μ-chip as chemical sensors (especially ethanol analyte) has been evaluated for measuring and detecting hazardous chemicals, which are not environmental affable. Improvement of doping of these nanosheets composed Sb_2_O_3_-ZnO MFs on μ-chip as chemical sensors is in the initial stage and no other reports are available. The MFs of Sb_2_O_3_-ZnO sensors have advantages such as stability in air, non-toxicity, chemical inertness, electrochemical activity, simplicity to assemble or fabrication, and bio-safe characteristics. As in the case of toxic ethanol sensors, the phenomenon of reason is that the current response in I-V method of Sb_2_O_3_-ZnO MFs considerably changes when aqueous ethanol are adsorbed. The calcined Sb_2_O_3_-ZnO MFs were applied for modification of chemical sensor, where ethanol was measured as target analyte. The fabricated-surface of Sb_2_O_3_-ZnO MFs sensor was made with conducting binders (EC & BCA) on the μ-chip surface, which is presented in the Figure 4(c–d). The fabricated μ-chip electrode was placed into the oven at low temperature (50.0°C) for 2 hours to make it dry, stable, and uniform the surface totally. I–V signals of chemical sensor are anticipated having Sb_2_O_3_-ZnO doped thin film as a function of current versus potential for hazardous ethanol. The real electrical responses of target ethanol are investigated by simple and reliable I–V technique using Sb_2_O_3_-ZnO MFs fabricated μ-chip, which is presented in Figure 4e.The time holding of electrometer was set for 1.0 sec. A significant amplification in the current response with applied potential is noticeably confirmed. The simple, reliable, possible reaction mechanism is generalized in Scheme 1d in presence of ethanol on Sb_2_O_3_-ZnO MFs sensor surfaces by I–V technique. The ethanol is converted to water and carbon dioxide in presence of doped nanomaterials by releasing electrons (-6e^−^) to the reaction system (conduction band, C.B.), which improved and enhanced the current responses against potential during the I–V measurement at room conditions.

**Figure 5:**
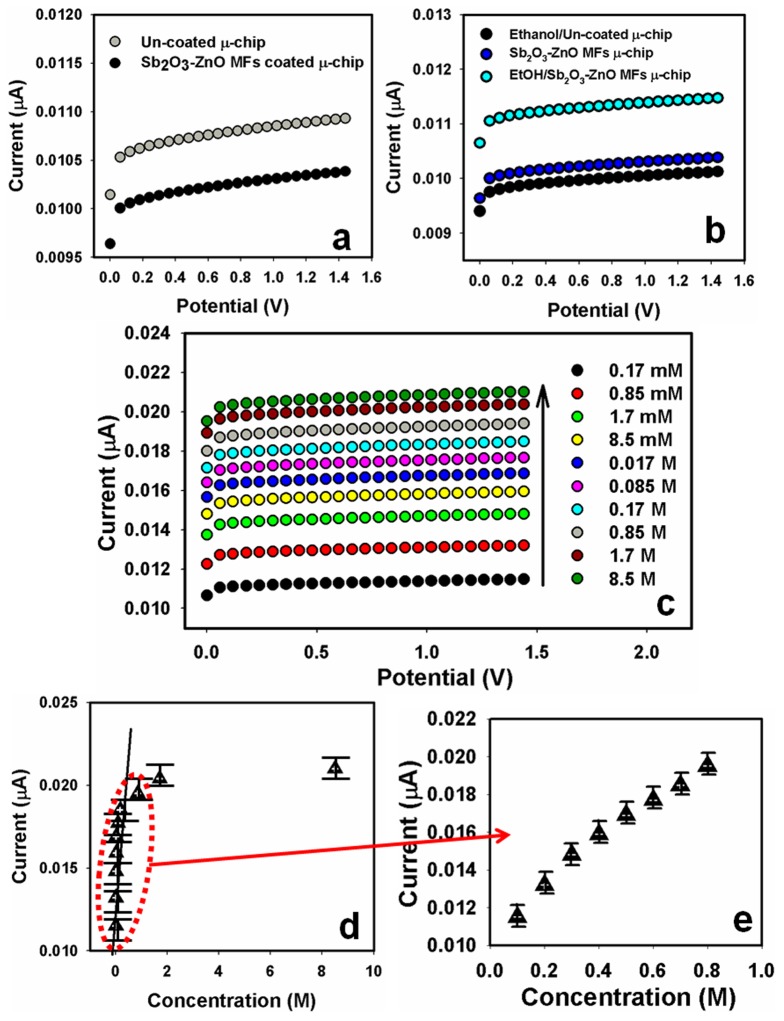
I–V responses of (a) un-coated and Sb_2_O_3_-ZnO MFs coated μ-chip without ethanol; (b) with 0.17 mM ethanol for un-coated μ-chip; without ethanol for Sb_2_O_3_-ZnO MFs coated μ-chip; and with 0.17 mM ethanol for Sb_2_O_3_-ZnO MFs coated μ-chip; (c) concentration variations (0.17 mM to 8.5 M) of analyte; and (d) calibration plot of doped nanomaterial fabricated on μ-chip surfaces. Potential was chosen in 0.1 to +1.4 V ranges. Error limit of I-V measurement was ± 0.001. There are three trial has been done in same experimental concentration at similar condition. Coefficient variation (CV): 0.1699.

Figure 5a shows the current responses of un-coated (gray-dotted) and coated (dark-dotted) μ-chip working electrodes with Sb_2_O_3_-ZnO MFs in absence of target ethanol. With nanosheets composed MFs fabricating surface, the current signal is slightly reduced compared to uncoated μ-chip surface, which indicates the surface is slightly blocked with doped MF nanomaterials in the buffer system. The current changes for the un-coated μ-chips (dark-dotted) towards target ethanol (∼50.0 µL), and MF nanomaterials modified film before (deep-blue-dotted) and after (light-blue-dotted) injecting of target 50.0 µL ethanol (∼0.17 mM) onto Sb_2_O_3_-ZnO MFs modified μ-chips is showed in Figure 5b. A significant current enhancement is exhibited with the Sb_2_O_3_-ZnO MFs modified μ-chips compared with uncoated μ-chips due to the presence of micro-structures, which has higher-specific surface area, larger-surface coverage, excellent absorption and adsorption capability into the porous MF surfaces towards the target ethanol. This significant change of surface current is examined in every injection of the target ethanol onto the doped modified μ-chips by electrometer. I–V responses with doped Sb_2_O_3_-ZnO MFs modified μ-chip surface are investigated from the various concentrations (0.17 mM to 8.5 M) of ethanol, which is showed in Figure 5c. It shows the current changes of fabricated μ-chip films as a function of ethanol concentration in room condition. It was also found that at low to high concentration of target analyte, the current responses were enhanced regularly. The potential current changes at lower to higher potential range (potential, +0.10 V to +1.3 V) based on various analyte concentration are observed, which is clearly presented in Figure 5c. A large range of analyte concentration is measured the probable analytical limit, which is calculated in 0.17 mM to 8.5 M. The calibration (at +0.3V) and magnified-calibration curves are plotted from the various ethanol concentrations, which are presented in the Figure 5(d–e). The sensitivity is estimated from the calibration curve, which is close to ∼5.848 µAcm^−2^mM^−1^. The linear dynamic range of this sensor displays from 0.17 mM to 0.85 M (linearity, R = 0.9989) and the detection limit was considered as 0.11±0.001 mM [3×noise (N)/slope(S)].

Usually, the resistance value of doped semiconductor materials are decreased with increasing surrounding active oxygen, which is the fundamental characteristics of nanomaterials [59]. Actually, oxygen adsorption demonstrates an significant responsibility in the electrical properties of the Sb_2_O_3_-ZnO MFs onto μ-chip. The oxygen ion adsorption is removed the conduction electrons and increased the resistance of Sb_2_O_3_-ZnO MFs. Unstable oxygen species (i.e., O_2_^−^ & O^−^) are adsorbed on the doped MF surface at room temperature, and the quantity of such chemisorbed oxygen species is directly depended on morphological and structural properties. At room condition, O_2_^−^ is chemisorbed, while on nanosheets composed microflowers morphology, O_2_^−^ and O^−^ are chemisorbed significantly. For this reason, the active O_2_^−^ is disappeared quickly [60]. Here, ethanol sensing mechanism on Sb_2_O_3_-ZnO MFs/μ-chip sensor is executed due to the presence of semiconductors oxides. The oxidation or reduction of the semiconductor MFs is held, according to the dissolved O_2_ in bulk-solution or surface-air of the neighboring atmosphere according to the following equations (vi–viii).

**O_2_**_(diss)_ (Sb_2_O_3_−ZnO MFs/μ-chip) **→ O_2_**_(ads)_ **(vi)**

**e^−^** (Sb_2_O_3_−ZnO MFs/μ-chip)**+O_2_ → O_2_^−^      (vii)**

**e^−^** (Sb_2_O_3_−ZnO MFs/μ-chip)+**O_2_^−^** → **2O^−^     (viii)**

These reactions are held in bulk-system or air/liquid interface or adjacent atmosphere due to the small carrier concentration which enhanced the resistances. The ethanol sensitivity could be attributed to the high oxygen deficiency on Sb_2_O_3_-ZnO MFs/μ-chip (eg. MO_x_) and higher density conducts to increase oxygen adsorption. Larger the quantity of oxygen adsorbed on the fabricated sensor surface, larger would be the oxidizing potential as well as faster would be the oxidation of ethanol. The reactivity of ethanol would have been very large as compared to other fabricated material surfaces surface under identical condition [61]–[63]. When ethanol reacts with the adsorbed oxygen on the exterior/interior of the Sb_2_O_3_-ZnO MFs/μ-chip layer, it oxidized to carbon dioxide and water by releasing free electrons (6e^−^) in the conduction band, which is expressed through the following reactions (ix).

**CH_3_CH_2_OH_(ads)_+6O^−^_(ads)_ →2CO_2_+3H_2_O+6e^−^(C.B.) (ix)**

**Figure 6:**
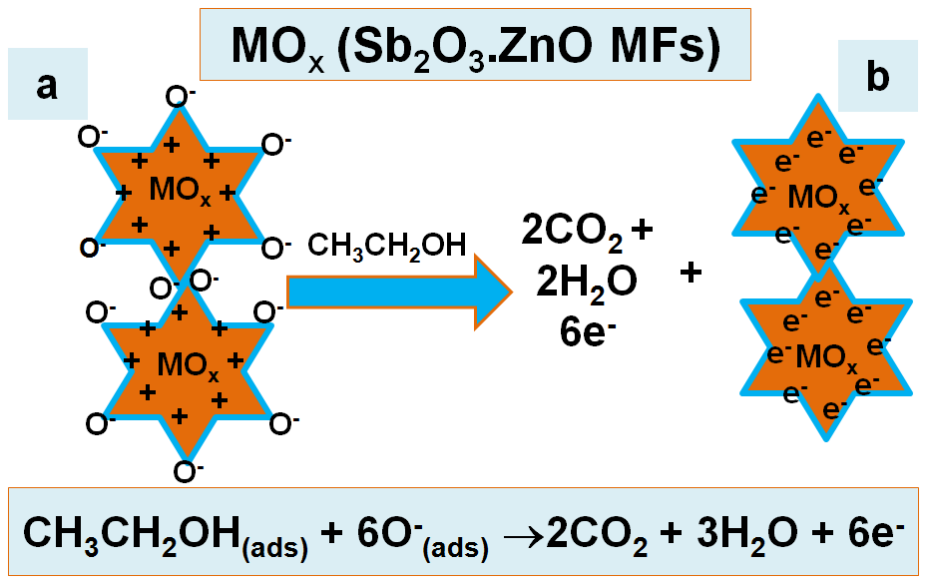
Mechanism of Sb_2_O_3_-ZnO MFs/μ-chip ethanol chemical sensors at ambient conditions.

In the reaction system, these reactions referred to oxidation of the reducing carriers. This method is enhanced the carrier concentration and consequently decreased the resistance on adjacent reducing analytes. The elimination of ionosorbed oxygen amplified the electron concentration onto Sb_2_O_3_-ZnO MFs/μ-chip and hence the surface conductance is increased in the film [64], [65]. The reducing analyte (ethanol) gives electrons to Sb_2_O_3_-ZnO MFs/μ-chip surface. Consequently, resistance is reduced, and hence the conductance is increased. This is the cause why the analyte response (current) amplifies with increasing potential. Thus produced electrons contribute to rapid increase in conductance of the thick Sb_2_O_3_-ZnO MFs/μ-chip film. The Sb_2_O_3_-ZnO MFs unusual regions dispersed on the surface would progress the capability of nanomaterial to absorb more oxygen species giving high resistance in air ambient, which is presented in Figure 6.

On the other approach, the utmost ethanol response of Sb_2_O_3_-ZnO MFs/μ-chip was attributed to the larger chemical communication on the sensing surface due to the larger surface area and meso-porous natures. The high ethanol response of the Sb_2_O_3_-ZnO MFs can be understandable in more detail in relative to the probable chemical-sensing mechanism, in terms of p-type doped semiconductor nano-materials. The oxide surface of an p-type semiconductor is readily covered with chemisorbed oxygen [66], [67]. Therefore, at identical condition, the adsorption of negatively charged oxygen can generate the holes for conduction. The subsequent ethanol-sensing reactions might be considered according to the charges of the adsorbed oxygen species (Sb_2_O_3_-ZnO MFs/μ-chip) under the statement of full oxidation of C_2_H_5_OH according to the following equations (x-xi).

**^1^/_2_O_2(g/l)_ ↔ O^−^_(ads)_** (Sb_2_O_3_−ZnO MFs/μ−chip)+**h**° **(x)**

**C_2_H_5_OH_(g)_+6O^−^_(ads)_+6h° → 2CO_2(g)_+3H_2_O_(g)_ (xi)**

The oxidation reaction with reducing ethanol amplifies the resistivity of the surface regions of the p-type doped Sb_2_O_3_-ZnO MFs/μ-chip, which in turn enhances the sensor resistance. The resistive contacts among the Sb_2_O_3_-ZnO MFs nano-materials control the chemi-sensor resistance. Therefore, the ethanol response is extensively dependent upon the dimensions of the nanosheet composed MFs, the large-active surface area and the nano-porosity. According to the charge accumulation reproduction of p-type semiconductors, the conduction occurs along the conductive as well as active sensor surface of Sb_2_O_3_-ZnO MFs/μ-chip [68]–[70].

The sensor response time was ∼10.0 sec for the Sb_2_O_3_-ZnO MFs coated μ-chip sensor to achieve saturated steady state current in I–V plots. The major sensitivity of μ-chip sensor can be attributed to the good absorption (porous surfaces MFs fabricated with binders), adsorption ability, high-catalytic activity, and good bio-compatibility of the Sb_2_O_3_-ZnO MFs/μ-chip. The expected sensitivity of the MF fabricated sensor is relatively better than previously reported ethanol sensors based on other composites or materials modified electrodes [71]. Due to perceptive surface area, here the doped nano-materials proposed a beneficial microenvironment for the toxic chemical detection (by adsorption) and recognition with excellent quantity. The prominent sensitivity of Sb_2_O_3_-ZnO MFs affords high electron communication features which improved the direct electron communication between the active sites of nano-sheets composed microstructures and μ-chips. The modified thin Sb_2_O_3_-ZnO MFs/μ-chip sensor film had a better reliability as well as stability in ambient conditions. Sb_2_O_3_-ZnO MFs/μ-chip exhibits several approaching in providing ethanol chemical based sensors, and encouraging improvement has been accomplished in the research section.

**Figure 7:**
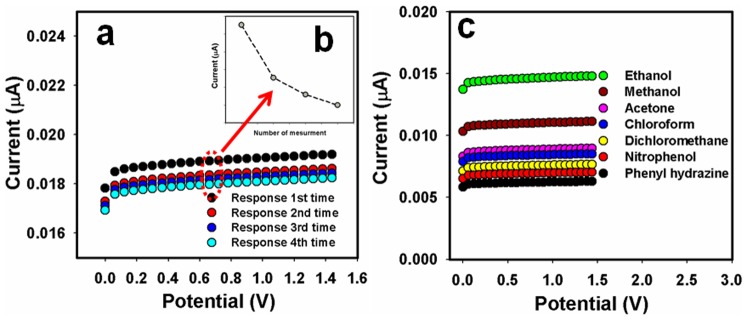
I–V responses of Sb_2_O_3_-ZnO MFs coated μ-chip are presented for ethanol sensors reproducibility (a), sensor-responses (inset, b), and selectivity (c) study. Ethanol and other chemicals concentration are taken as1.0 mM for selectivity study.

To check the reproducibly and storage stabilities, I–V response for Sb_2_O_3_-ZnO MFs coated μ-chip sensor was examined (up to 2 weeks). After each experiment, the fabricated Sb_2_O_3_-ZnO MFs/μ-chip substrate was washed thoroughly with the PBS buffer solution and observed that the current response was not significantly decreased (Figure 7a). The sensitivity was retained almost same of initial sensitivity up to week (1^st^ to 2^nd^ week), after that the response of the fabricated electrode gradually decreased. A series of six successive measurements of 0.17 mM ethanol in 0.1 mM PBS yielded a good reproducible signal at Sb_2_O_3_-ZnO MFs/μ-chip sensor in different conditions with a relative standard deviation (RSD) of 3.7% (Figure not shown). The sensor-to-sensor and run-to-run repeatability for 0.17 mM ethanol detection were found to be 1.9% using Sb_2_O_3_-ZnO MFs/μ-chip. To investigate the long-term storage stabilities, the response for the MFs sensor was determined with the respect to the storing time. The long-term storing stability of the Sb_2_O_3_-ZnO MFs/μ-chip sensor was investigated significantly at room conditions. The sensitivity retained 94% of initial sensitivity for several days. The above results clearly suggested that the fabricated sensor can be used for several weeks without any significant loss in sensitivity. The dynamic response (0.17 mM to 0.85 M) of the sensor was investigated from the practical concentration variation curve. The sensor response time is mentioned and investigated using this sensor system at room conditions. In this study, it shows the MFs/Chips-based ethanol sensor response after successive injection in buffer solutions containing 1.0 mM of ethanol, which is presented in Figure 7a. A recovery time is observed with a small loss of signal (∼0.007uA), suggesting that the sensor can be re-used further [Figure 7b (inset)]. It was also investigated the sensing selectivity performances (interferences) with other chemicals like methanol, acetone, chloroform, dichloromethane, phenyl hydrazine, nitrophenol etc. Ethanol exhibited the maximum current response by I–V system using MFs fabricated micro-chip electrode compared to methanol and others. It was specific towards ethanol compared to all other chemicals. A comparative study with all chemicals using Sb_2_O_3_-ZnO MFs/μ-chip is included in Figure 7c. In Table 1, it is compared the performances for ethanol chemical detection based Sb_2_O_3_-ZnO MFs/μ-chip using various modified electrode materials [72]–[80].

**Table 1:** Comparison the performances for ethanol detection based on various nanomaterial fabricated electrodes.

Materials	Methods	Linear Dynamic Range, LDR	Sensitivity	Linearity, r^2^	Limit of Detection, LOD	Response Time	References
**Ni/Pt/Ti**	Potential amperometry	–	3.08 µAmM^−1^ cm^−2^	–	–	–	[72]
**Ni-doped SnO_2_ nanostructure**	I–V	1.0 nM∼1.0 mM	2.3148 µA cm^−2^mM^−1^	0.8440	0.6 nM	10.0 s	[73]
**Pd–Ni/SiNWs electrode**	Potential amperometry	–	0.76 mAmM^−1^cm^−2^	0.9970	10.0 µM	–	[74]
**ZnO-CeO_2_ Nanoparticles**	I–V	1.7 mM∼1.7 M	2.1949 µA cm^−2^mM^−1^	0.9463	0.6±0.05 mM	10.0 s	[75]
**RuO-modified Ni electrode**	Cyclic voltammetry	100∼1000 ppm	4.92 µAppm^−1^cm^−2^	–	–	13.0 s	[76]
**CeO_2_ nanoparticles**	I–V	0.17 mM∼0.17 M	0.92 µAcm^−2^mM^−1^	0.7458	0.124±0.010 mM	10.0 s	[77]
**Al-doped ZnO nanomaterial**	I–V method	Up to 3000 ppm	1000 ppm ethanol	–	–	∼8.0 s	[78]
**CuO nanosheets**	I–V	up to 1.7 M	∼0.9722 µAcm^−2^mM^−1^	0.7806	0.143 mM	10.0 s	[79]
**Sm-Doped Co_3_O_4_ Nanokernels**	I–V	1.0 nM∼10.0 mM	2.1991±0.10 µAcm^−2^mM^−1^	0.9065	0.63±0.02 nM	10.0 s	[80]
**Sb_2_O_3_-ZnO MFs**	**I–V**	**0.17 mM∼0.85 M**	**5.845 uA cm^−2^mM^−1^**	**0.9989**	**0.11±0.02 mM**	**10.0 s**	**Current work**

## Conclusion

Transition-metal doped semiconductor Sb_2_O_3_-ZnO MFs are prepared by easy, simple, efficient, reliable, and economical approaches using reducing agents. The structural, morphological, and optical properties are performed by using XRD, XPS, FE-SEM, and UV-visible techniques respectively. The Sb_2_O_3_-ZnO MFs/μ-chip has assembled by simple fabricated method and displayed higher sensitivity for chemical sensing. They are efficiently prepared for sensitive ethanol sensor based on Sb_2_O_3_-ZnO MFs embedded μ-chips with conducting coating binders, for the first time. The analytical performances of the fabricated ethanol MFs sensors are excellent in terms of sensitivity, detection limit, linear dynamic ranges, and in short response time. Sb_2_O_3_-ZnO MFs/μ-chips are exhibited higher-sensitivity (∼5.845uA cm^−2^mM^−1^) and lower-detection limit (∼0.11±0.02 mM) with good linearity in short response time, which efficiently utilized as chemi-sensor for ethanol onto μ-chips. This novel attempt is introduced a well-organized route of efficient chemical sensor development for environmental toxic pollutants and health-care fields in broad scale.
